# Treatment of tibia avulsion fracture of posterior cruciate ligament with high-strength suture fixation under arthroscopy

**DOI:** 10.1007/s00068-015-0606-9

**Published:** 2015-12-11

**Authors:** W. Zhu, W. Lu, J. Cui, L. Peng, Y. Ou, H. Li, H. Liu, W. You, D. Wang, Y. Zeng

**Affiliations:** 1 Department of Sports Medicine, Shenzhen Second People’s Hospital (First Affiliated Hospital of Shenzhen University), 518035 Shenzhen, Guangdong People’s Republic of China; 2 Shenzhen Tissue Engineering Laboratory, 518000 Shenzhen, People’s Republic of China; 3 Shenzhen Sports Medicine Engineering Laboratory, 518000 Shenzhen, People’s Republic of China; 4 Guangzhou Medical University, 510182 Guangzhou, People’s Republic of China; 5 Biomechanics and Medical Information Institute, Beijing University of Technology, 100022 Beijing, People’s Republic of China

**Keywords:** Avulsion fracture, Posterior cruciate ligament, Arthroscopy, High-strength line

## Abstract

**Aim:**

To evaluate the outcome of arthroscopy treatment using high-strength line in the treatment of tibial avulsion fracture of posterior cruciate ligament.

**Methods:**

Both the avulsed bone block and the tibia bone bed were refreshed. The procedure was completed with the assistance of PCL director drill guide. The reduction and fixation using high-strength line were used to fix the avulsed bone by from posterior middle portal. Rehabilitation began early postoperatively.

**Results:**

From January 2010 to June 2012, a total of 18 arthroscopically treated cases of PCL tibial avulsion fracture were retrospectively evaluated. Reduction of the avulsion fragment was obtained in all cases. 16 cases were followed up for 7–30 months (average 13.6), and 2 cases were out of follow-up. In the 16 followed patients, flexion and extension were back to normal within 6 weeks, and return to normal walk in 12 weeks. The bone healing was good without any vascular or nerve complications. All the patients regained the preinjury activity level. The mean score (and standard deviation) increased from 38.9 ± 4.9 points to 95.2 ± 3.8 points with the system of Lysholm, from 57.1 ± 10.3 points to 94.3 ± 4.4 points with the system of IKDC. Post-test displacement of KT3000 declined from 3.6 ± 0.39 to 1.1 ± 0.27 mm.

**Conclusion:**

Arthroscopic vertical fixation by high-strength line is a simple, safe, reliable, and micro-invasive treatment to PCL tibial avulsion fracture. It is a kind of real all arthroscopic technique, and good for early postoperative rehabilitation. The total stability of the knee could be gained, and the second operation to remove the internal fixation is avoided.

## Background

Posterior cruciate ligament is an important structure to maintain the stability of the knee joint. The damage on the posterior cruciate ligament directly results in the posterior instability of the knee joint, as well as the backward translocation of the patella and the patella ligament. The stress on the cartilage in the joint increases and the interventricular load conduction is disturbed, resulting in the degenerative changes of the joint. The reduction and internal fixation of avulsion fracture at the distal insertion of the posterior cruciate ligament under direct vision have good effects, but the operative invasion is extensive [1]. Recently, the arthroscopic surgery technology has undergone important advances [2]. The reduction and fixation with the assistance of arthroscopy have the advantages of small invasiveness and quick recovery. But the operation is difficult under the arthroscope, and the fixation effects are potentially unstable [3]. From January 2010 to June 2012, the method of fracture reduction under arthroscope and fixation by high-strength suture was adopted. The results indicated good reduction, firm fixation, and quick recovery. The report is as follows. We hypothesized that arthroscopic vertical fixation by high-strength line is a simple, safe, reliable, and micro-invasive treatment to PCL tibial avulsion fracture.

## Materials and methods

### Case information

**Fig. 1:**
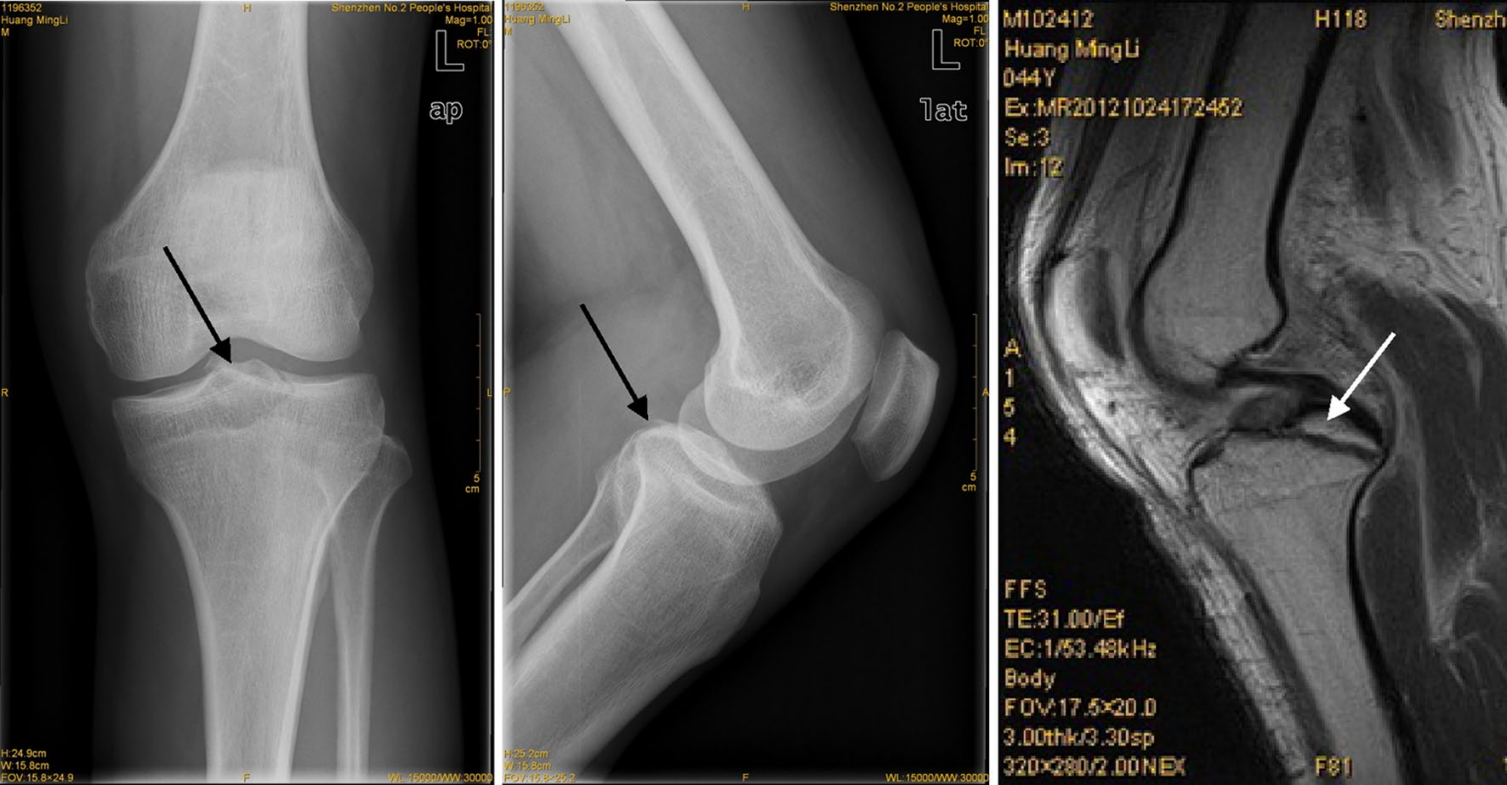
Preoperative X-ray and MRI examinations. Preoperative X-ray and MRI examinations indicate the avulsion fracture at the distal insertion of the posterior cruciate ligament (represented by the *arrow*)

From January 2010 to June 2012, 18 cases of avulsion fracture at the distal insertion of posterior cruciate ligament were admitted to our department. They were all treated with the method of reduction and freshing under the arthroscope and fixation by knotting the high-strength suture around the posterior cruciate ligament. The duration of the disease before the surgery was 1–3 weeks. All the patients had the history of acute trauma at the knee joint. The result of posterior drawer test was positive. The examinations by CT scan, X-ray, and MRI showed avulsed fragment shadow at the back of tibia (Fig. 1). Before operation, the Lysholm scores [4] were 38.9 ± 4.9; IKDC scores were 57.1 ± 10.3; and the backward displacement value was 3.6 ± 0.39 mm by KT3000. All the operations were conducted by the same experienced surgeon in sports medicine. In the 18 cases, 12 were male and 6 were female; the age was 21–48 years, with the average of 31.6. Ten cases had avulsion fracture on the left knee and 8 on the right knee. All the cases were caused by direct force resulting in the avulsion fracture at the distal insertion of the posterior cruciate ligament. Five cases were combined with meniscus injuries; three cases were combined with medial collateral ligament injury (one case underwent reconstruction and 2 cases received conservative treatment).

### Surgery methods

**Fig. 2:**
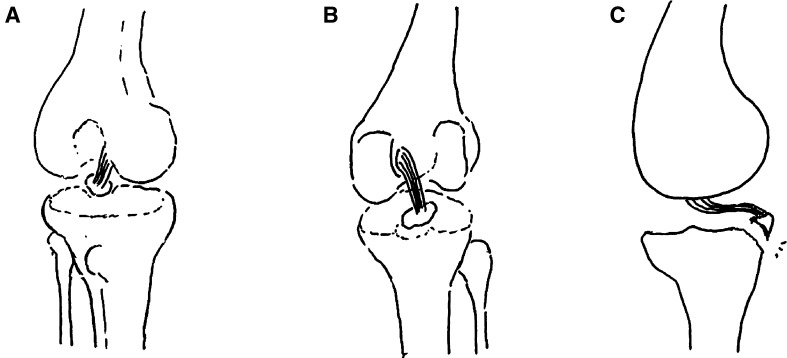
Preoperative schematic drawings. Preoperative schematic drawings indicate the avulsion fracture at the distal insertion of the posterior cruciate ligament. **a** Anteroposterior position schematic drawing. **b** Posteroanterior position schematic drawing. **c** Lateral schematic drawing

**Fig. 3:**
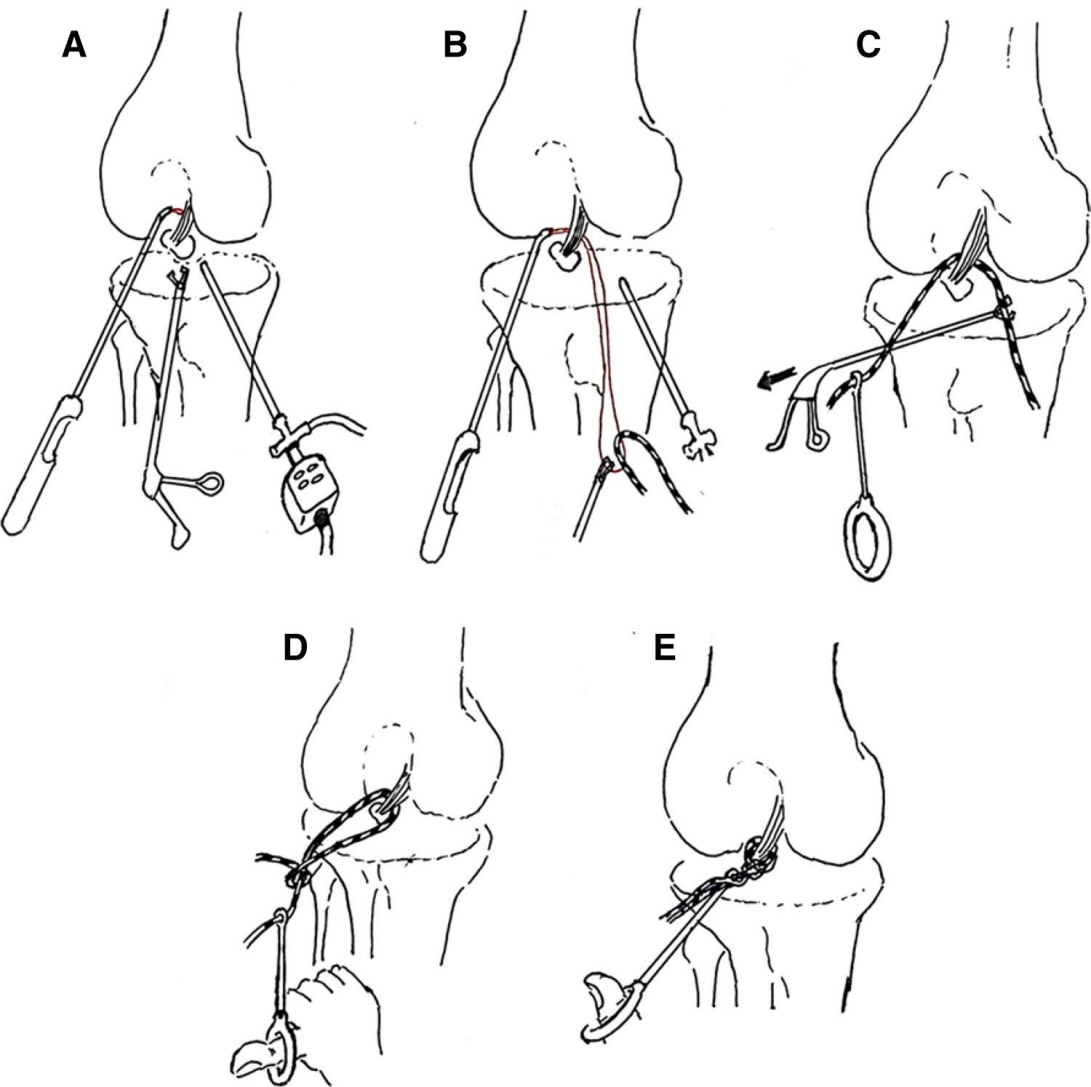
Operative schematic drawings. Operative schematic drawings indicate that the suture passer for rotator cuff suture was used to place high-strength suture around the distal insertion of the posterior cruciate ligament and knotted. **a** Retrieve the PDS suture from the suture passer with a suture retriever. **b** Place a high-strength suture around the loop of the PDS suture. **c** Retrieve the PDS suture with the high-strength suture and make the high-strength suture around the distal insertion of the posterior cruciate ligament. Retrieve the other end of the high-strength suture through the same portal. **d**, **e**, Use the knot pusher to knot at the distal insertion of the posterior cruciate ligament

**Fig. 4:**
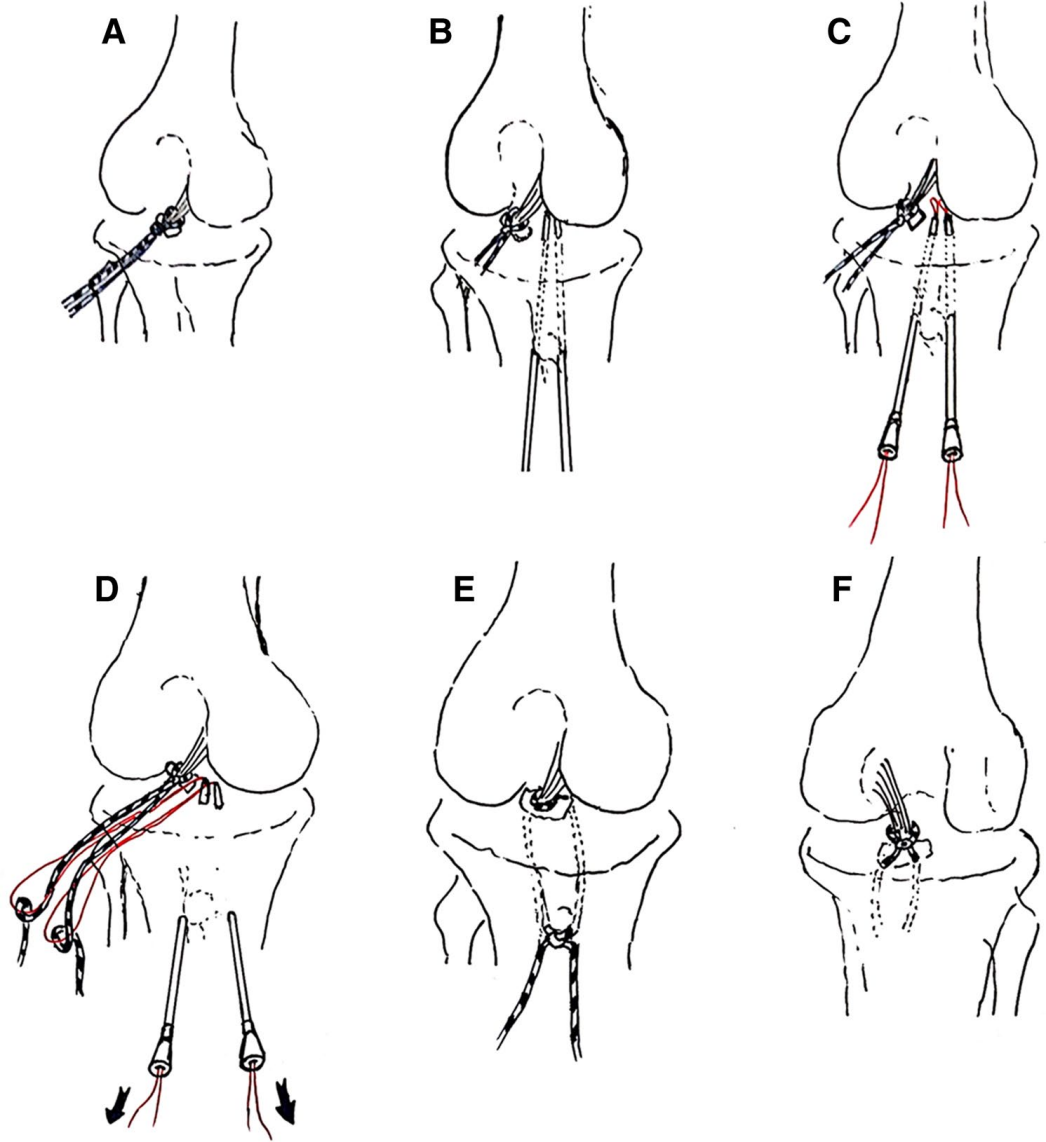
Operative schematic drawings. Operative schematic drawings indicate the high-strength suture around the distal insertion of the posterior cruciate ligament passing through the bone tunnels. **a** The distal insertion of the posterior cruciate ligament with a high-strength suture knot on it. **b** A Kirschner wire with the diameter of 2.0 mm was drilled from the lateral area of the tubercles of tibia to the inferolateral area of the fragment. The other Kirschner wire with the diameter of 2.0 mm was drilled from the medial area of the tubercles of tibia to the inferomedial area of the fragment. **c**, **d** The PDS lines inserted through two lumbar puncture needles were extracted from the two bone tunnel to the front of the tubercles of tibia. **e** The high-strength suture was tightened and tied at the front of the tubercles of tibia (anteroposterior position schematic drawing). **f** The high-strength suture was tightened and tied at the front of the tubercles of tibia (posteroanterior position schematic drawing)

**Fig. 5:**
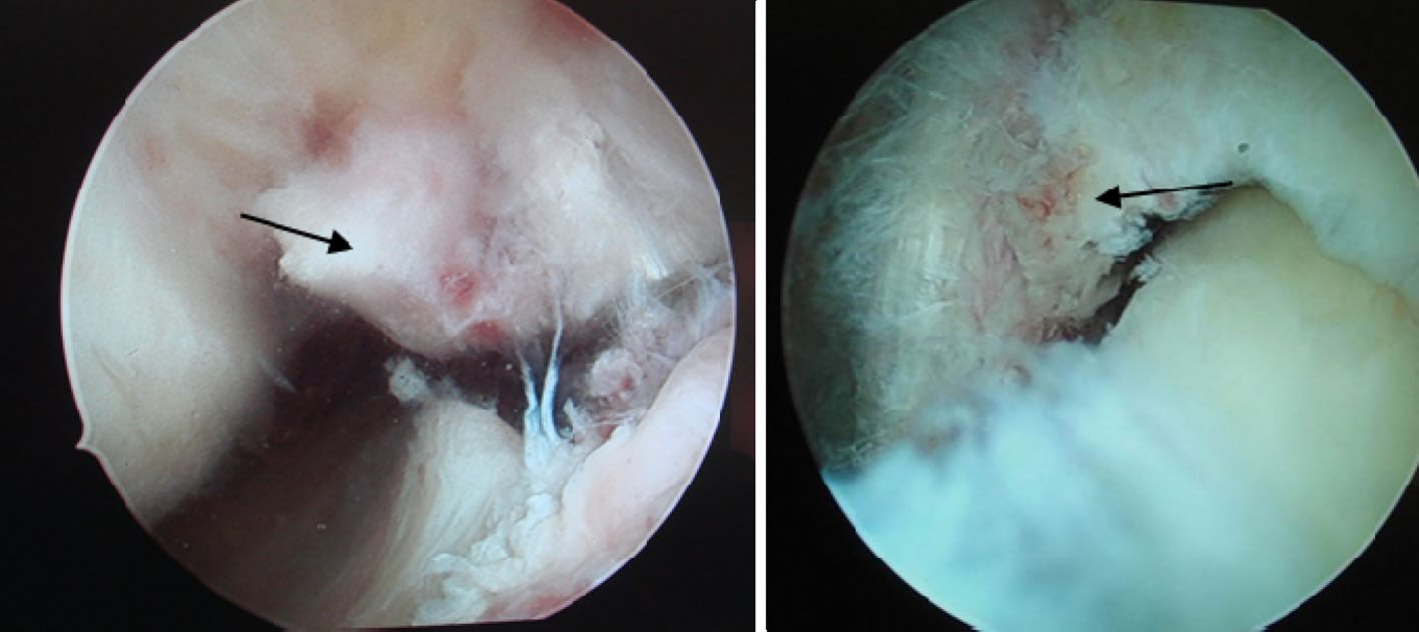
Preoperative observation under arthroscope. The avulsion at the distal insertion of the posterior cruciate ligament under the arthroscope (Fig. 5 shows the anterior interior approach and Fig. 5 the posterior exterior approach)

**Fig. 6:**
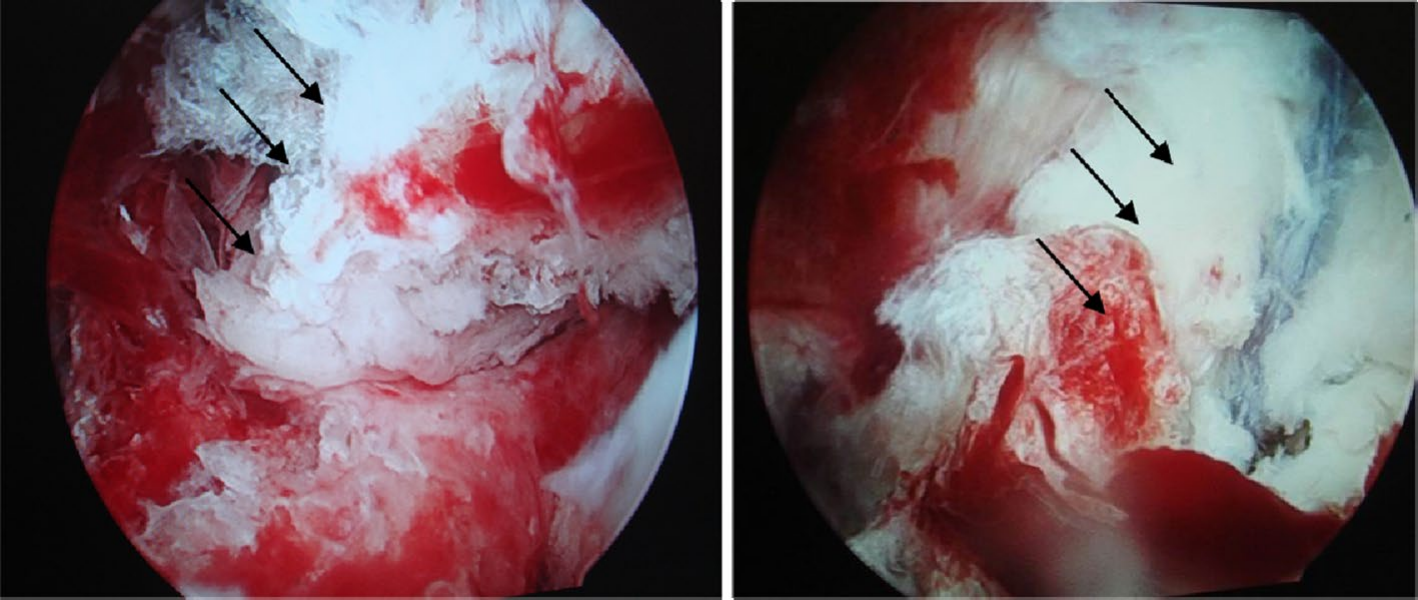
Preoperative and postoperative observation under arthroscope. The preoperative and postoperative status under the arthroscope (Fig. 6 shows the preoperative status and Fig. 6 the postoperative status)

The patient was placed in supine position and received general anesthesia or combined with lumbar epidural anesthesia. The approaches at the knee joint were marked. Under the pneumatic tourniquet of 300 mmHg, the anteromedial and anterolateral approaches were used to examine the knee joint and to determine the lesion (Fig. 1a). The arthroscope and equipments were inserted via the posterior exterior and posterior interior approaches to remove the soft tissues around the fracture fragments, so that the bone blocks were only attached to the posterior cruciate ligament (Fig. 2b, c). The fracture blocks and the lateral bone bed of tibia were freshed. The tissues impeding the reduction were removed. The reduction was achieved with the hook. The suture passer (produced by Smith & Nephew Inc.) for rotator cuff suture was used to place two high-strength suture(produced by Smith & Nephew Inc.) around the distal insertion of the posterior cruciate ligament and knotted (Fig. 3). The guide for posterior cruciate ligament reconstruction was inserted via the anterior interior approach past the inferomedial side of posterior cruciate ligament. The reduction was performed under the arthroscope. Then the guide for posterior cruciate ligament reconstruction was inserted via the anterior interior approach past on the inferomedial area of the posterior cruciate ligament. Under the arthroscope, a Kirschner wire with the diameter of 2.0 mm was drilled on the inferomedial area of the fragment. The aiming device for posterior cruciate ligament was inserted via the anterolateral approach past on the lateroinferior area of the posterior cruciate ligament. Under the arthroscope, a Kirschner wire with the diameter of 2.0 mm was drilled on the lateroinferior area of the fracture block (Fig. 4b). The drilling sites of the Kirschner wires were at the both sides of the tubercles of tibia. The two Kirschner wires were used to drill a hollow borer with the diameter of 4.5 mm under the arthroscope. A PDS suture shuttle was inserted through the hollow borer. They were extracted with a cramp for further use (Fig. 4c, d). The PDS lines inserted by the method described above were extracted from the two bone tunnel to the front of the tubercles of tibia. The high-strength suture was tightened. The fracture reduction status was inspected. The high-strength suture was tightened and tied at the front of the tubercles of tibia (Fig. 4e, f). The firmness of fixation was checked by knee flexion and extension under the arthroscope (Figs. 5, 6). Other combined injuries were treated subsequently.

### Postoperative recovery

After the operation, the knee was bandaged with cotton bandage and fixed by the knee-brace. The muscle exercises of the muscular systems on the lower limbs began 24 h after the surgery. Three days after the surgery, the exercise of joint flexion and extension by 0°–90° began with CPM machine. Under the protection of braces, the patients used crutches to support part of the weight. The active flexion by 90° was realized 6 weeks after the surgery, and the flexion and extension functions were recovered 8 weeks after the surgery. X-ray scan and CT scan were carried out every 2–3 weeks after the surgery to check the bone blocks. The patients began walking with full weight-bearing with knee-brace 6 weeks after the surgery and recovered to the preoperative situation 12 weeks after the surgery. The use of knee-brace should not be shorter than 3 months.

### Postoperative follow-up

The Lysholm and IKDC scoring systems were used to evaluate the postoperative recovery of the knee joint functions after. The KT3000 was used to evaluate the laxity of the knee joint. The results were analyzed with SPSS14.0. Difference with *P* < 0.05 was regarded as statistically significant.

## Results

The surgery lasted for 35–55 min. No nerve or blood vessel injuries occurred. During the follow-up, two patients were missed. The follow-up was successful for 16 cases. The follow-up duration was 7 months to 30 months, with the average of 13.6 months. One patient showed difficulty of self-recovery. Manipulation under anesthesia was conducted 6 weeks after the surgery and the knee recovered to the preinjury flexion and extension angles. The flexion and extension functions of 15 patients were normal and recovered to the preinjury status. The Lysholm scores of the 16 follow-up patients increased from preoperative 38.9 ± 4.9 to postoperative 95.2 ± 3.8; the IKDC scores increased from 57.1 ± 10.3 to 94.3 ± 4.4; and the KT3000 scores dropped from 3.6 ± 0.39 to 1.1 ± 0.27 mm. The differences between preoperative and postoperative values were all significant (*P* < 0.05).

**Fig. 7:**
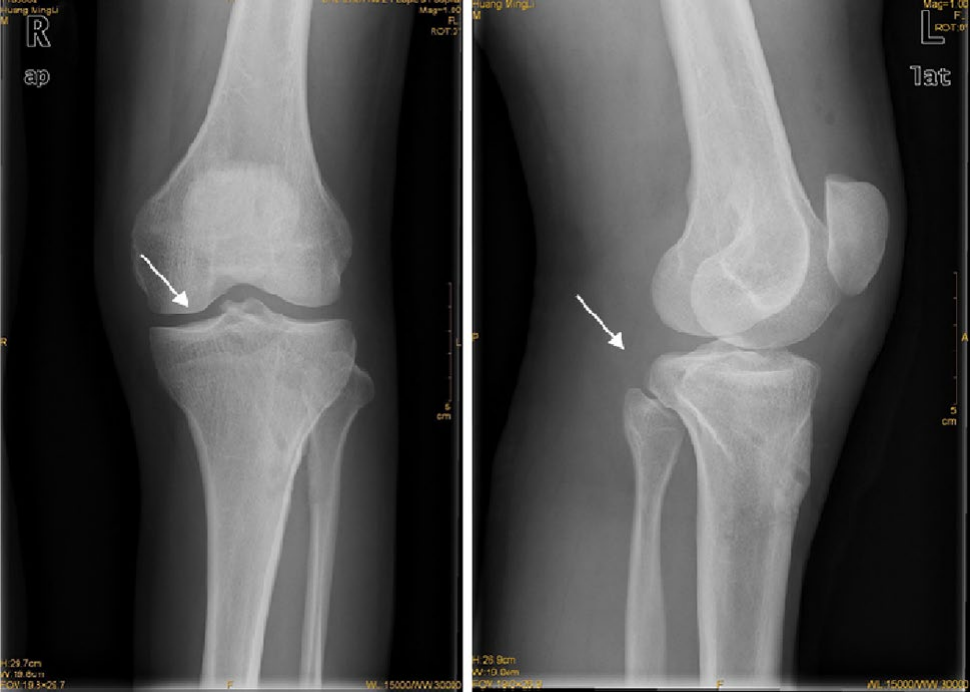
Postoperative X-ray. Postoperative X-ray shows good avulsion fracture reduction at the distal insertion of the posterior cruciate ligament (represented by the *arrow*)

**Fig. 8:**
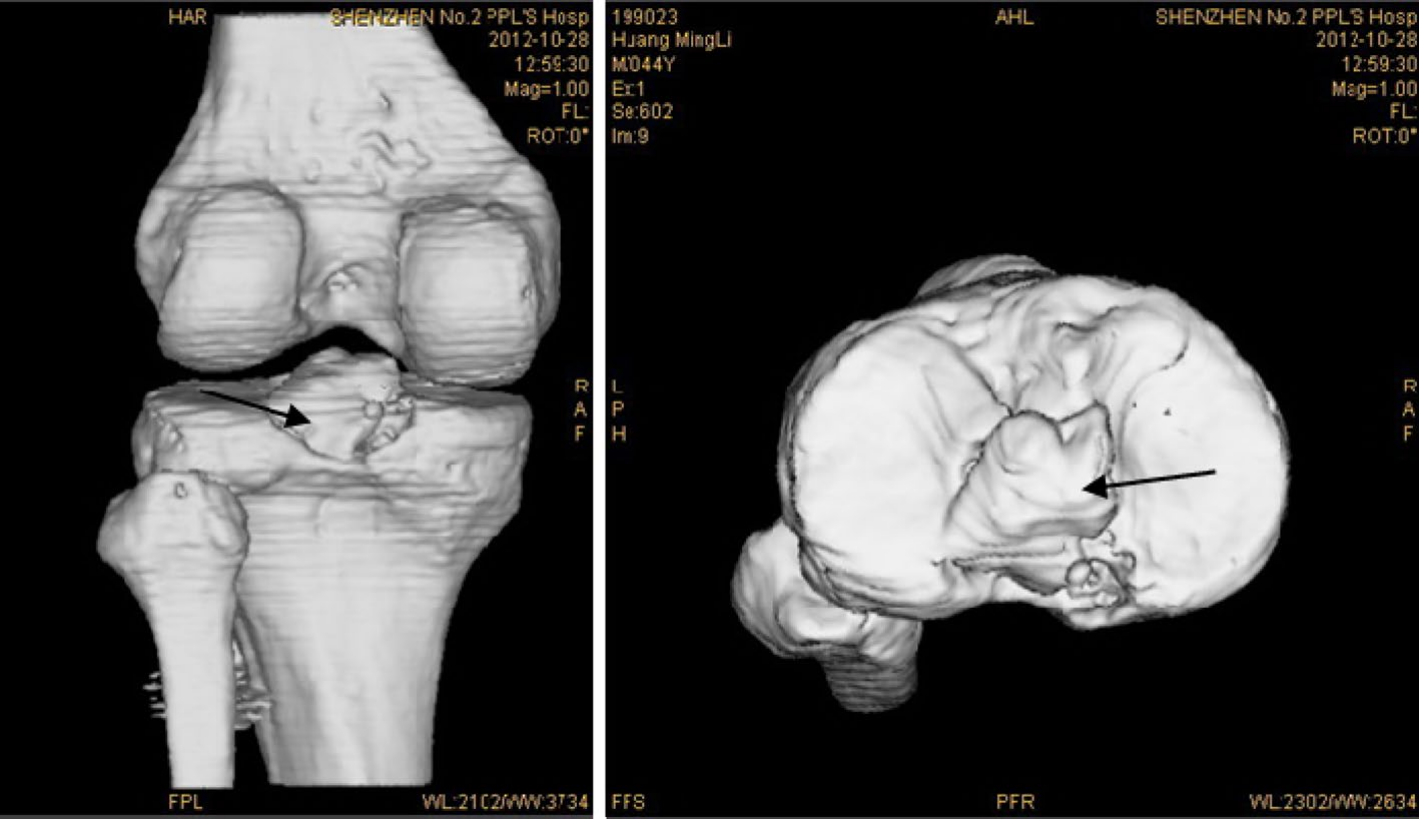
Postoperative three-dimensional reconstructed CT scan. Postoperative three-dimensional reconstructed CT scan shows good fixation and reduction at the distal insertion of the posterior cruciate ligament (represented by the *arrow*)

The X-ray examination and three-dimensional reconstructed CT scanning showed a satisfying reduction for the 16 patients. The fracture was all recovered 3 months after the surgery. All the follow-up patients were satisfied with the results (Figs. 7, 8).

## Discussion

With the progress in the research on the anatomy, physiological functions, and biomechanism of the posterior cruciate ligament, the posterior cruciate ligament injuries draw an increasing attention [5]. Especially with the development of the arthroscopy technology in recent years, the diagnosis and recovery of posterior cruciate ligament injuries and reconstruction under the arthroscope have undergone rapid development [6]. The avulsion fracture at the distal insertion of the posterior cruciate ligament is a special type of the posterior cruciate ligament injuries. The cases are not rare [7]. Inappropriate treatment could cause instability of the knee joint, which leads to the degenerative changes of the knee joint. Except that the fracture with large fracture fragment without displacement can be treated with plaster fixation, surgery should be conducted to the displaced fractures to reconstruct the facet of the knee joint [8]. In the past, posterior approach under direct vision was often used for the fracture reduction and screw fixation. The fixation effects are good, but the invasiveness is considerable and the operation process is difficult to handle. The screws could cause bone splitting of the avulsion fragment. The recovery process is long and the chance for postoperative adhesions is huge. So the function recovery is not satisfying [9].

This research used the fixation by high-strength suture under the arthroscope. The follow-up investigation indicated satisfying clinical effects. The advantages are as follows: (1) The fracture fixation has good effect; although the high-strength fixation is a soft fixation, the high-strength suture has high mechanical strength and the suture has a certain flexibility. The bone fragments are allowed slight movements, which conforms to the principles of bio-fixation. (2) During minimal invasion, the reduction and internal fixation do not need an incision. The surgery is done with several small incisions. Under the arthroscope, the vision is clear, preventing the injuries to nerves and blood vessels effectively. The postoperative recovery is quick, and early-stage recovery is possible. (3) It is suitable for cases combined with structural injuries such as meniscus and medial collateral ligament injuries. (4) Secondary surgery is not necessary, reducing the economic burden for the patients.

During the surgery process, the following aspects should be noted under the arthroscope: (1) The posterior cruciate ligament attachment to the tibia is located in the lacuna at the posterior, medial side of the tibia. The posterior joint cavity is small and irregular. The popliteal nerves and blood vessels are attached closely to the posterior. Therefore, interior and exterior approaches should be established and all the operations should be conducted under the surveillance of the arthroscope, to prevent injuries to blood vessels and injuries. (2) When the 4.5-mm bone tunnel is being drilled, the curet should be used to support the tip of the Kirschner wire, to prevent the backward rotation of the Kirschner wire with the 4.5 mm drill bit, which would otherwise damage the blood vessels and nerves. (3) In bone bed freshing, the granulation tissue on the bone bed should be removed thoroughly, or the reduction results will be poor. (4) During the bone bed freshing and guide insertion process, normal ligaments and cartilages should be protected. (5) Preoperative training should be established to reduce the operation time, or the pneumatic tourniquet should be loosened during the surgery, causing hemorrhage inside the joint. The vision could be fuzzy, causing difficulties for the operation and damaging the blood vessels and nerves. This study has several limitations. The number of patients included was small. But we believe that these data would be valuable for evaluating the outcomes of arthroscopy treatment using high-strength line in the treatment of tibial avulsion fracture of posterior cruciate ligament.

The effects of all the cases were satisfactory. No complications such as the injuries to the blood vessels and nerves occurred. The fixation effect similar to that in open reduction was achieved and the recovery duration was shortened. Patients soon resumed normal life and work. The method is direct; the operation time is short, the damage is minimal, and the recovery is quick. It is worthy of wide application. The operation under arthroscope is complicated and requires excellent skills. Therefore, it should be practiced by surgeons with adeptness at arthroscopic operation and rich experience, as well as complete anatomical knowledge, in order to increase the clinical effects of the surgery and to prevent complications.

## References

[1] Frosch K, Proksch N, Preiss A (2012). Treatment of bony avulsions of the posterior cruciate ligament (PCL) by a minimally invasive dorsal approach. Oper Orthop Traumatol..

[2] Zhang X, Cai G, Xu J (2013). Wang K.A minimally invasive postero-medial approach with suture anchors for isolated tibial avulsion fracture of the posterior cruciate ligament. Knee..

[3] Chen CW, Chen L, Pan ZE, Yang SW (2012). Open reduction and internal fixation via a posterior approach for posterior fractures of tibial plateau. Zhongguo Gu Shang..

[4] Lyshom J, Gillquist J (1982). Evaluation of knee ligament surgery results with special emphasis onuse of a scoring scale [J]. Am J Sports Med.

[5] Andri GT, Klineberg EO, Wahl CJ, Mills WJ (2008). Treatment of posterior cruciate ligament tibial avulsion fractures through a modified open posterior approach: operative technique and 12- to 48-month outcomes. J Orthop Trauma..

[6] Sasaki SU, da Mota e Albuquerque RF, Amatuzzi MM, Pereira CA (2007). Open screw fixation versus arthroscopic suture fixation of tibial posterior cruciate ligament avulsion injuries: a mechanical comparison. Arthroscopy..

[7] Wajsfisz A, Makridis KG, Van Den Steene JY (2012). Fixation of posterior cruciate ligament avulsion fracture with the use of a suspensory fixation. Knee Surg Sports Traumatol Arthrosc..

[8] Shelbourne KD, Urch SE, Freeman H (2011). Outcomes after arthroscopic excision of the bony prominence in the treatment of tibial spine avulsion fractures. Arthroscopy..

[9] Hoogervorst P, Gardeniers JW, Moret-Wever S (2010). Pseudo-arthrosis repair of a posterior cruciate ligament avulsion fracture. Knee Surg Sports Traumatol Arthrosc..

